# Practical Illustrations of Typhus Fever, of the Common Continued Fever, and of Inflammatory Diseases, &c. &c.

**Published:** 1820-01-01

**Authors:** 


*Practical Illustrations of Typhus Fever, of the common continued Fever, and of Inflammatory Diseases, &c. &c.* By John Armstrong, M. D. Physician to the Fever Institution of London. Third Edition. One Volume Octavo, 566 pages. London, 1819.

In the Review of the First Edition of this admirable Book in our Monthly Series, a very full account of the practical part of Dr. Armstrong's work will be found; we shall, therefore, in this place, take the liberty of making some alteration in the author's arrangement, and shall treat the subject in a more general way than that which he has thought proper to adopt.

The title of "Practical Illustrations of Typhus, &c." by no means describes the valuable mass of information which follows. No doubt, Dr. Armstrong intended to write only a practical essay upon typhus; but his work speedily, and inevitably, became a disquisition upon the pathology of fever in general, illustrated by a practical account of some of the various modifications of that disease. If Dr. A. had foreseen this, he would, perhaps, have put his thoughts into a more systematic, and, therefore, more intelligible form; but after all, we believe that the present plan was in some degree the best.

Writers of systems have been dealt with very unjustly; no sooner have some of their leading principles been proved to be untrue, than their, perhaps otherwise, splendid publications have been consigned to the dusty shelf, although the very fact of illustrating a theory presupposes that the writer has collected together a large mass of valuable observations; and even such might have been the fate of Dr. A's patient and most accurate investigations into the symptoms and treatment of disease. For, as system after system has fallen before the increasing precision of medical reasoning, it would be too sanguine to suppose, that the present explanations of the phenomena of fever, are to leave nothing for future discoverers to perform. It is indeed perfectly apparent, that they are defective in many points, as we shall attempt to shew; but we feel bound to say, that the work before us, raises its excellent author to the very highest rank of medical philosophers; conspicuous as it is for cautious deduction, for freedom from hypothetical leaning, for accurate and most extended observance of morbid phenomena, and for, we were going to say, unrivalled vividness of description. Although Dr. A. has not thought proper to give his work the garb of a theoretical disquisition, yet, as his explanations involve a complete revolution in the generally received doctrines of fever, we shall attempt to place before our readers an outline of the theory of fever, which Dr. A. has, either directly or indirectly, suggested to our minds, and then proceed to illustrate that outline by an analysis of the invaluable practical descriptions of disease and modes of treatment with which his volume abounds.

Little or nothing was formerly known of the pathology of febrile diseases; and yet the state of fever forms the whole, or a part of almost every complaint to whose attack the human frame is liable; for, until very lately, if not at the present moment, one of the chief indications of cure, as taught at all the schools where Dr. Cullen's nosology is used, was thus stated: "To obviate the tendency to death"—this means, that we were so entirely unacquainted with the nature and essence of fever, that we cured our patients by moderating the symptoms, and, if possible, preserving life, until the disease should terminate by natural means. Let us add to this, that the doctrines of Cullen led, in other ways, to the most erroneous practice, and we shall find reason, on a comparison with the present work, to say much more in praise of the latter, than we think advisable to express in this place. We do not mean to say, that this good work has been accomplished solely by Dr. A.; the path had been gradually cleared by many accurate observers of nature; practical men, such as Dr. Hamilton,* had obeyed the impulse of reason in their treatment of disease; whilst others, with more extended powers of reasoning, had, with the same facts, laid the foundation of an improved pathology; much impression had also been made by the publication of numerous cases and dissections by the medical officers of the army and navy; but it was reserved for Dr. A. by the greatly increased accuracy of his discrimination in the different states of fever, to raise an edifice, the superstructure of which may be modified and improved, but whose basis, we believe, will be as immoveable as the facts upon which it is founded.

To estimate properly the improvements which Dr. A. has made in our knowledge of the pathology of fever, it

---

* Hamilton on purgatives.

will be necessary to recollect, what was the state of medical opinion on that subject previous to the publication of the first edition of his work.

At that time the theory of Cullen was principally taught in the scools; but a theory so entirely hypothetical as this, was forgotten by all sensible men in a short time after they began to apply their knowledge in the portals of disease; many practised without a theory, and, following only the dictates of their own good sense, bled, or purged, or did both, just as chance, or other motives, had led them to adopt either method, for the cure of the state of excitement which they saw before them: others, in their post mortem examinations, finding the most evident traces of previous inflammation, concluded, that the essence of fever consisted in an inflammation, particularly of the brain, and treated their patients accordingly; but it was in vain to look for any rational explanation of the phenomena of fever, in indiscriminate practice like this. The symptoms which, in all fevers, or nearly so, precede the stage of excitement and inflammation, were overlooked, or were not attended to, by all; and, consequently, a whole tribe of fevers in which this secondary stage of excitement does not exist, or is only partially exhibited, was passed over and neglected. In both ancient and modern medical writings indeed, we find cases described, in which the body remained oppressed, so that the stage of excitement did not occur; but as our author observes, "only obscure hints are to be found in the writings of others respecting the pathology of this variety of fever;" [p. 69.] in short, such cases, when they occurred, were considered to be anomalous, and were not understood.

It is in giving a distinct account of this oppressed or, as he calls it, congestive state of fever, that one of Dr. A's chief claims to our gratitude rests; and in thus enunciating it to our readers, it becomes a pleasing as well as most useful part of our duty, to trace, by what degrees, Dr. A. was led to engraft upon this discovery an arrangement of fevers, more simple and more perfect, than any which has been heretofore presented to the public. What improvements may we not expect, when such patience and acuteness as Dr. A's, in observing the phenomena of disease, are joined with an accurate examination of the body after death!

We are told, that evident marks of arterial fullness, or of previous inflammation are found on the dissection of persons dying of fevers in which the usual symptoms of increased heat and morbid excitement were present; but on examining the bodies of those dying of fever, in which the skin continued to be, from the first, pale and cold, the energies of the system nearly extinguished, and in which no symptom of excitement had occurred, the author invariably found, that the right side of the heart and the large veins were gorged with black grumous blood, and that not the slightest appearance of arterial fullness existed. Now, if we recollect with our author, that the necessary course of most, or probably all fevers, consists of a cold stage, or stage of oppression; a hot stage, or one of excitement; and a stage of decline or collapse, we shall be led to see, that, as in the stage of oppression, the blood is congested in the venous system; and in the stage of excitement a corresponding accumulation of blood takes place in the arterial capillaries, the fever is produced by some cause, which, probably by inducing debility of the heart, determines the blood into the venous system; and that the consequent excitement only occurs from the reaction of the constitution, to equalize the deranged circulation, by freeing itself from the load by which it is oppressed. It may be said, indeed, that Cullen's theory described the hot stage of fever as an effort of nature; but to relieve what?—Not the venous congestion which dissection shows to be present; but a spasm of the extreme arteries which existed no where but in the professor's imagination. Hence, no practical advantage, but the reverse, arose from this theory; for as by spasm was meant a state of debility, fevers were considered and treated as diseases of debility throughout. It will be sufficient to say of the inflammatory theorists, that they neglected the stage of oppression altogether, and mistook the effect of fever for the cause which produced it; for from the above simple account of the essential phenomena of fever, it must be evident, that the only necessary and primary part of the disease, falsely called fever, is an oppressed and actually debilitated state of the heart and muscular powers.

What produces this state? and how is it produced? The nervous system is the part by which the body receives impression from external agents; the body feels the effect of these impressions from the connexions of its nerves; these connexions are either with the muscles or with the heart. Motion is produced by the connexion of the nerves with the muscular system; whilst, through the medium of the heart, the nervous system gives birth to all the other actions of the body, healthy and diseased. So, if sudden debility of the nervous system be induced, the heart and moving powers will become debilitated in a corresponding degree; and such probably is the primary and only essential state of the body in fever. The cause of fever acting upon the nervous system produces debility; the heart shares in this debility; and hence the blood is accumulated in the venous system. The debilitating cause may act so violently as to produce death; if not, accumulation takes place in the veins, and the palpable symptoms of fevers arise in the order above mentioned.

In the volume before us, Dr. A. has, in several places, stated his conviction that the causes of fever act through the medium of the nervous system; but he has not distinctly described the mode in which he conceives the effect to be produced. Content with pointing out the practical differences of appearance which are found in the several stages, and seeing in the great majority of fevers, namely, those in which the stage of excitement exists, that the practical pathology of the disease refers chiefly to the state of the vascular system, he has been apparently led to neglect the nervous system, by paying too exclusive an attention to the heart and arteries, in his account of the disease; but although he has perhaps expressed himself, occasionally, in an unguardedly strong way, still the neglect is only an apparent one. When once the symptoms have been laid in train by the primary effect upon the nervous system, the disease, for almost all practical purposes, becomes a disease of the vascular system; all our remedies are directed through that system; in short, as we yet possess no power of appreciating the disorders which take place in the nerves, or perhaps of directly obviating them, when they have taken place, the nervous system becomes a very secondary matter for consideration. The time may come, when our present methods of curing diseases of excitement and inflammation will be entirely changed; when our present imperfect, because indirect, efforts by bleeding and other antiphlogistic means, will be changed for the application of some simple remedy, which, by acting directly upon the nervous system, will cure diseases, with a rapidity and a certainty, of which we can at present have no conception.

The medical philosopher would have been thankful to Dr. A. if he had been more explicit upon this abstruse, yet most interesting part of the inquiry. It is to be hoped, however, that he will hereafter investigate more closely the part which the nervous system performs in the drama of living actions; and from the rare combination which his mind affords of caution, acuteness, and unbiassed simplicity of object, we may perhaps live to see, practically, that the hints we have here thrown out, are founded upon a proper anticipation of what is to happen.

Led by these grand distinctive marks between the different states and stages of fever, Dr. A. has divided fevers into the simple, the inflammatory, and the congestive fevers.

In simple fever, the debilitating shock which the system has sustained, by the cause producing the disease, is not sufficient to destroy life; the stage of oppression runs its course, reaction takes place, the blood is gradually transferred from the venous to the arterial system, and the stage of excitement is established. The venous congestion is thus entirely removed; and as the organization is peculiarly perfect, or from some other favourable circumstance, a state of simple increased action is produced, in which there exists no obstruction to the circulation of the blood; and consequently no inflammation. The blood only runs its round of the circulation with increased rapidity; for, with little exception, this circumstance constitutes the whole of that part of the disease. After a time the excitement declines, and the third stage, of collapse, supervenes; all increased action subsides, and the patient is either left weak but convalescent, or he dies, probably more from "an exhaustion of the vital principle induced by the preceding excitation," [15] than from disorganization of any part of the body; for we only find that "dissection commonly reveals some remains of an injected state of the capillary arteries, without any effusion of coagulable lymph, adhesion of parts, gangrene or suppuration, which are the results of genuine inflammation." 15.

Such is simple fever. But let the loss of balance between the arteries and veins be greater; let the venous congestion be more concentrated; let some one part of the body be weakened, or otherwise so disorganized as to offer obstruction in that part to the increased impetus of the arterial circulation; and inflammation takes place. In this way, an entirely new feature is given to the disease; new sets of symptoms arise, according to the part which is ininflamed; the danger is aggravated, and after death, "effusion of coagulable lymph, adhesions, suppuration, and occasionally some approaches even to gangrene itself,"[34] are found in the brain. The effects of inflammation are also found in the medulla spinalis; or similar disorganizations occur in the chest or abdomen, according to the peculiar viscera which had suffered, from the inflammation having been particularly seated in them.

In this way, the fever puts on the inflammatory type; or, the first shock, causing the fever, is so violent as nearly to exhaust the energies of life; or the constitution of the patient is particularly weak, or some other still uninvestigated circumstance is present, so that the reaction does not take place, or is only partially developed, and the heart and circulation are depressed; the skin is pale and cold, the secretions are suspended, the whole body is oppressed, as with a load, there is much anxiety of the precordia, and either the excitement is but partially developed, or it is entirely wanting. The blood, on being drawn, does not show the buffy coat; and on dissecting the body after death, the large veins in the viscera and the right side of the heart are gorged with black grumous blood. By these signs the congestive fever may be known.

Thus we see, that " in the simple excitive and inflammatory forms of fever, the action of the heart and arteries is increased; but in the congestive form it is diminished: and this difference in the action of the heart, together with the high temperature of the two former, and the low temperature of the latter, constitutes the most distinct mark between diseases of excitement and congestion." [87] Again, " the venous system is more immediately and chiefly concerned in the phenomena of the congestive typhus, and the arterial system in those of the simple and inflammatory typhus" [84]; also, " the congestive differs from the simple typhus, first, because the viscera are far more engorged in the primary stage; and secondly, because, through the continuance of the engorgement, that stage is followed by a general collapse, without the intermediate one of regular and universal excitement; which not only partly characterizes the simple typhus, but which produces the occasional and partial congestions of the last stage. If, then, the congestive so obviously differs from the simple, it may be inquired, in what does it differ from the inflammatory typhus? Universal augmentation of heat, and excitement, attend the inflammatory typhus, which are not the concomitants of the true congestive typhus; and may be considered as the principal *external* distinctions between them. But, further, there is in the inflammatory, not only a general excitement of the arteries, but an increased a cumulation of blood in the capillaries of the dise sed part; whereas, in the congestive, the force of the arterial system is not only diminished generally, but the whole venous circulation oppressed, and particularly obstructed, where the congestion occurs." 82.

We have thus given an outline of the great facts from which Dr. A. has deduced his arrangement, and we have also, in a rather imperfect manner, sketched out the theory of fever which results from its adoption; but, before laying before our readers, a more detailed account of the excellent descriptions of disease, and methods of treatment, with which this volume is filled, we shall still further consider the subject in a theoretical point of view, and especially remark in what particulars it appears to be deficient or erroneous. In the first place, various states of fever suggest themselves, which seem to be difficult of explanation by the above doctrine. We allude to the many kinds of remitting fevers, and indeed to the slight remissions of symptoms which are found to take place in all fevers, except such as hurry their victim to an untimely grave, with uncontroled and violent rapidity; but especially, we allude to the whole class of intermittents; and here we must, *in limine*, remark, that with regard to the last-mentioned tribe of fevers, the doctrine that inflammation of a particular organ may form a part of the disease, is absolutely untenable, for they are evidently simple fevers, each paroxysm forming a distinct and perfect fever, the constitution being only left in a state fit to be acted upon by a fresh recurrence of the nervous condition, upon which the attack depends. In this case, the stage of perspiration, by taking off the excited action of the arterial system in the stage of excitement, resolves the fever, in a manner, similar to the change which takes place in the stage of collapse of ordinary fever, except that in the latter case the system is left debilitated by the long continued excitement, or congested state of the veins, or from some of its important parts being more or less disorganized by the inflammatory action which has preceded. In this way, we consider that intermittent fevers afford a beautiful illustration of the general truth of the doctrine here set forth; especially also when we recollect, that these fevers become continued, when the violence of their symptoms increase; that is, when that small addition of inflammation takes place, which we consider to be essential even to the stage of excitement in simple continued fever, (otherwise, we think they would intermit) or the more intense arterial engorgement of particular parts happens, which characterizes the state of inflammatory fever as above described. Such fevers again intermit, when the sub-inflammatory action, constituting the simple fever, subsides. We do not include the second and more intense state of inflammatory action; because we believe, that in such cases, the fevers seldom or never take upon themselves again the true intermitting type.*

The question of remitting fevers we consider as explicable upon the grounds, that when the excitement, or inflammation connected with them is not very violent, the increased action is occasionally diminished for a time, by the occurrence of perspiration, or of some other exciting, *i. e.* depleting action. Should the above explanation be deemed satisfactory, the same mode of reasoning will apply to the fevers of children, which are generally transient, or remitting; because the general state of uninjured soundness which their organs present, gives little room for the action of those causes of obstruction which, in ordinary fevers, produce inflammation and its consequences.

Although we think that the above cases offer no objection to the doctrine of fever under discussion, we believe that it is at least defective when applied to the explanation of several very principal occurrences in fever. We know too little of the state of the parts in cases of inflammation, to make the explanation, here given of the difference between simple and inflammatory fevers, especially when applied to the numberless varieties of shades of difference which occur, little more than a probable assumption. We shall, however, give here, as some further hint at elucidation, our own extension of Dr. A's statement of the difference between simple excitement and inflammation, in page 21 of his work. Both excitement and inflammation involve increased action of the large vessels; but in the former, the circulation is free, though increased; in the latter, obstruction takes place in the capillary arteries, and the disease of inflammation is produced. Pain occurs from the pressure upon the nerves by swelling; redness, from

* Following up this view of the subject, it may perhaps be deduced that the intermittent is the only pure specimen of simple fever, and that Dr. A's simple fever is only less complicated with inflammatory or sub-inflammatory action, than his order of inflammatory fever; or the continued type of his simple fever may be kept up in common fever, by the existence of some action which is not distinctly inflammatory, but which is very different from simple excitement; or, in contagious fevers, by the altered state of the blood which, sooner or later, forms a part of such cases.

the accumulation of blood, and perhaps from its being forced into the *white* vessels; throbbing, from the increased pulsatory pressure, and consequent pain, caused by the action of the heart behind; and thus the symptoms go on, until the obstruction becomes complete, when gangrene takes place, or the action subsides, and the parts recover their wonted powers, or the secreting vessels take upon themselves a new action; and being assisted by the absorbents, pus is formed. But neither of this last process, nor of what constitutes the primary obstruction, can we offer any explanation.

As a further objection to the doctrine, we may say, that we know nothing of the mode in which the causes of fever produce their effects upon the nervous system; nor what those effects are: nothing, or very little, of the peculiarities of that debility of the vascular system which produces congestion of the veins; almost nothing, in fact, of the precise mode in which any one of the phenomena of fever is produced by those which precede it. Again, Dr. A. thinks that there are various fevers in which no previous state of oppression existed; but which are diseases of excitement from the commencement, such as those transitory increases of heat, &c. which follow either the local irritation of a blow, or of a surgical operation, or "the direct application of a stimulus not sufficient at once to inflame any particular part, but to excite the heart into increased action, and the heat of the body beyond the common standard," as "exposure to an elevated temperature, the use of ardent spirits, wine, or the like, strong mental emotions, rich food, and excessive exercise;" "the local affections which are so apt to supervene being generally to be traced to the increased action of the heart operating on local predispositions, as already explained; these predispositions chiefly varying according to climate, habits, and hereditary structure." 34.

Although the production of fever, in this way, does not appear to be theoretically impossible; yet we confess, we are inclined to believe, that in many, if not in all of the above-mentioned cases, there is a state of oppression and congestion. Such a state evidently follows many blows and operations, when they occasion fever; and we see no reason why the stroke of the sun, improper excess of any kind, or strong mental emotion, should not as readily produce a debilitating effect upon the nervous system, and consequently a stage of oppression, as contagions, marsh effluvia, or vicissitudes of the weather; and if so, may not this stage have been overlooked, even by so accurate an observer as Dr. Armstrong?

We have still a few more observations to make on the first cause of fever, and the state of the body during that first period, which we consider as the only essential and *invariable phenomenon* of fever, except, indeed, in the instances above spoken of, if they be really exceptions.

We hold, somewhat in contradiction to the words, though, we believe, not to the real opinion of Dr. A. that the primary state of the body in fever, namely, that which is followed by the venous congestion, is a state of real debility. If so, the essence of fever is debility, in contradiction to the letter of Dr. A's expression, that "an extensive observation during a series of years, has convinced me, that the genuine typhus, so far from being of an asthenic character, is, most certainly, an affection of excitement or of congestion, in its first stages, requiring the evacuant plan." [8] Again, "in the first stage of the simple typhus the debility is merely apparent, and chiefly dependent upon the preternatural accumulation of blood in the veins." [18] We grant that the congestive stage, very often, perhaps always, requires evacuating remedies; but still we contend, that before symptoms of reaction have occurred, the constitution is in a state of direct debility, and that such state is the only essential part of the disease called fever. Let it never be forgotten, however, that we agree most fully to the fact, that in all cases, after the development of excitement, the treatment is purely antiphlogistic; and that it is apparently so, even in the stage of oppression, in many cases. But we are now upon points of accuracy, and we think that such accuracy may ultimately turn out to be of the greatest importance. Why may not we be, some time or other, in possession of a stimulating remedy, which, by being applied to the system, immediately on the production of the very first stage, may renovate the impaired energies of the constitution, and thus prevent the formation of the fever altogether? Nay, have not stimulants, accidentally or designedly applied at such a time, even the stimulants of Dr. Brown, produced this very effect? Is it not, in short, the natural robust state of the constitution which preserves the body from fever, in the thousands of cases in which the causes of fever are applied without its being produced? and does not the want of what is called the predisposing causes of fever, mean only, that the body is in possession of this natural mode of preventing or curing the action of the debilitating cause which, under other circumstances, produces fever? But we forbear to proceed.

The experienced reader will readily perceive how great an alteration this doctrine of fever will, if true, make in our nosological arrangements. To use the words of our author,

"The pyrexiæ of Nosologists shall therefore be considered as a class, in which three orders are comprehended; two orders in which the causes are specific, though at the same time essentially different; and one order in which the causes are not specific, but common. The first order comprehends fevers proceeding from contagions; the second order, fevers proceeding from marsh and similar miasmata; the third, fevers proceeding from the vicissitudes of the weather and other ordinary causes," such as "the general impression of heat or cold, local irritations, or any cause not having the special properties of contagion, or of marsh effluvium." 3.

In this arrangement, the greatest departure from our usual notions occurs in the new idea which is here given us of the phlegmasiæ; so new is it, indeed, that we have not yet quite convinced ourselves of its propriety. The simple acute inflammations are thus placed in the class of fevers; whereas, in common cases, these diseases put on so little of the character of fevers, that we can scarcely reconcile the change to ourselves. The difficulty consists in this—when pneumonia, for instance, is joined with typhus, or any fever arising from specific contagion, the whole complaint is so much altered in its appearance, that the affection of the chest becomes evidently subordinate; and indeed, in these cases, the fever is so predisposed to run a certain course, that though we cure the pneumonia, still the fever will go on for a certain time afterwards, which does not occur in the fevers arising from common causes; for in them, when we have cured the pneumonia, the fever ceases.

Whether this circumstance arises from any peculiarity in the nature of the operative cause of fevers, or only arises from the mere duration of the disease, produced by contagion, we are at a loss to determine. Sure we are, however, that it is one of much importance in practice, if it do not hereafter form a proper basis for such fevers, and those which arise from common causes, being considered as distinct classes of disease nosologically speaking. In a practical point of view, it is of consequence, as it obliges us to be more cautious in the use of our evacuating practice, when the inflammations are connected with contagious fevers, than in ordinary cases; since in the former, the constitution has to contend against the remaining term of the fever after the inflammation has subsided. Are the same precautions necessary in cases of marsh fevers? Our experience, in such cases, will not authorise us to answer the question.*

We have before mentioned, that we are not quite satisfied with the plan adopted by Dr. A. in the composition of his book. He has chosen to develope his principles gradually, whilst describing the symptoms and treatment of typhus fever; by which plan, he has indirectly exalted this fever to a situation which it ought not to hold. For, in fact, though it is one of the most formidable diseases with which the British practitioner has to contend, yet we must only consider it as one genus of fevers, differing principally from the rest, in the nature of the cause producing it; and therefore, before analysing the practical part of the work, we have thought it right to set forth a connected view of fever, considered as a class. We shall now, however, gladly proceed to illustrate what has been said, by a reference to Dr. A's able descriptions of typhus in some of its numberless states.

"In this Essay, the word typhus shall be limited to the peculiar disease which is allowed to originate from a specific contagion, and which doubtless has the power of producing an affection of its own nature in individuals exposed to its influence." 7.—"It is unquestionably most prevalent in cold or temperate climates," [8] "being in England evidently favoured by a low temperature," as it "prevails most in the cold seasons of winter and spring, and generally abates or disappears as the summer advances; whilst it often prevails to a considerable extent in cold wet autumns." 9.

"In a number of persons exposed to the contagion of typhus, some, though rarely, are attacked so early as the first or second

---

* We suspect that they are; for, as we shall presently show, we consider that the fixed term of fevers arising from specific contagion, forms no part of the effect primarily produced by the contagion; but that such fevers only run a certain course, when, from accidental circumstances, arising afterwards, such as the eruptions of small-pox and measles, and the altered condition of the blood in typhus, a state of constitution is produced which requires a certain time for its conversion again into the natural state. So the irritation upon the skin keeps up the fever in scarlatina, and the altered blood in typhus, even after inflammation may have been removed. Why may not the blood therefore be similarly altered in fevers from marsh miasmata, or even from common causes? We may perhaps expect it to become so in all cases where the fever is of sufficient length to allow of it; and if so, no such effect would happen in the acute phlegmasiæ, because the disease is usually so rapid in its progress, that it must be cured, or it will destroy the patient, before the blood has had time to become altered in its constitution, whilst an explanation would be given of the fact, that even typhus fevers are cut short, when properly treated *at the commencement.*

day, and others even after the thirtieth; but perhaps the most common periods for sickening after exposure are from the end of the first to the middle of the third week. It has been affirmed, that it follows at so great a distance as the ninth or tenth week after exposure; but this seems very questionable." 9.

It is evidently of the greatest importance to acquire a clear idea of the course of an attack of simple fever. We shall, therefore, extract Dr. A's description of simple typhus entire.

### *The simple Typhus.*

" The simple typhus has a first stage of oppression, a second of excitement, and a third of collapse. These successive stages, but more particularly the two last, bear a pretty exact ratio to each other as to degree, but not as to duration. The stage of oppression is usually marked by a variety of symptoms; among which, the following are mostly conspicuous. Paleness of the face; a peculiar look of dejection and of weariness; some degree of darkness or livor in the integuments surrounding the eyes; prostration of strength; diminution of mental energy and of sensibility; cold, creeping sensations on the surface, or short hot and chilly fits alternately; loathing of food; nausea or vomiting; whitish or clammy tongue; sense of weight or anxiety about the precordia; occasional sighing and hurried breathing; aching, heaviness, or giddiness of the head; coldness of the back, and pains of the loins; a quick, low, struggling pulse, changeable as to frequency, and even irregular as to force. These symptoms are accompanied with feelings of general uneasiness, somewhat resembling those which are experienced after a long journey, or any other great fatigue. The stage above described sometimes comes on and reveals itself with rapidity; but generally, it is more insidious in its approaches, and occupies from first to last a period of two or three days; when, after various irregular demonstrations of reaction, it is succeeded by the second stage, or that of excitement, in which there is a complete development of the fever. In subjects who possess constitutional vigour, the tone and velocity of the circulation are now preternaturally increased, and the pulse, accordingly, becomes comparatively expansive, thrilly, and somewhat resisting; at least it is widely different from the variable, confined, inelastic pulse of the former stage, and from the uniform free and smoothly-flowing one of health. The cheeks are flushed with a dusky redness; the eyes heavy, and the lips parched. The respiration is quick; the skin almost invariably dry; the heat universally diffused, and steadily above the common point; the tongue foul; the thirst urgent; the uneasiness in the head increased; the sensorium in a highly susceptible state; every symptom, in fine, denoting an excess of excitement. This second stage of the simple typhus, naturally holds a tolerably even tenour for some time. As it proceeds, however, the brain, at intervals, is usually disturbed with reverie or slight delirium, coming on towards evening, when there is an exacerbation of the fever; and receding towards morning, when there is a remission; but the prostration of strength, which is at all times very evident, is generally greatest in the period of exacerbations, and the tongue is then drier. During the predominance of the excitement, the bowels have, for the most part, a tendency to constipation. The excretions, as well as secretions, also undergo gradual and material changes, which are evinced by the dark and offensive nature of the fæces, by the peculiar odour of the breath and whole body, and by the morbid appearances exhibited on the tongue, in the fluids formed from the liver, from the kidneys, and from other organs of secretion.

"After six or seven days, sooner or later, according to its mildness, or severity, the stage of excitement gradually gives place to that of collapse; which is first announced by signs of depression in the voluntary powers; by a certain degree of relaxation in the skin; by a more variable and less concentrated state of the temperature; and by a notable diminution in the force of the circulation, the pulse being of less volume, softer and undulating. In the mildest cases, the approach of the state of collapse may be viewed as an indication of convalescence. For, although the patient may complain of much general weakness, and sometimes of soreness in the flesh, with flying pains or cramps in the extremities, yet the tongue will be found softer and cleaner, the thirst diminished, the pulse slower, the breathing deeper and less frequent, and the skin of a natural warmth as well as moisture. Besides, the patient will pass much better nights, the functions of the stomach will be in some degree restored, with an evident improvement in the appearance of the fæces, and in general, with a lateritious sediment in the urine. Whereas, in the more marked instances of this sort of typhus, the supervention of the stage of collapse considerably augments the danger. The prostration of strength then becomes far greater; the pulse is commonly quicker, and always much weaker; the tongue fouler, darker, and drier; the voice fainter, and the articulation less distinct; the respiration short, or feebler, and more anxious. The sensorial functions too are more disordered, and the countenance is more dejected, sunk, and inanimate. Added to these symptoms, the skin feels looser, and appears more shrivelled, while the temperature is no where so intense as in the stage of excitement, but variable in the course of the day, even on the central parts; and there is an increase of general restlessness, a more perceptible and peculiar fœtor about the body, and often an irritating species of cough, which comes as it were in convulsive fits. In this state the patient is disposed to lie on his back. As the peril increases, he not only labours under subsultus tendinum, visual deceptions, low muttering delirium, and difficulty of deglutition, but has also a tendency to slide downwards in the bed, and to draw up the feet frequently towards the body." 10.

The simple typhus generally terminates favourably, but when mortal from neglect or maltreatment, "dissection commonly reveals an injected state of the capillary arteteries, without any effusion of coagulable lymph, adhesion of parts, gangrene or suppuration, which are the results of genuine inflammation," [15.] but there is probably always some degree of lesion in the structure of some vital organ, though the morbid change may elude the inquisition of the anatomist.

In this excellent description of simple typhus, how plainly do we recognize the stages of oppression, of excitement and collapse, passing gradually into each other and forming a whole, "the pathology of which is applicable to almost all the mildest forms of other fevers; for, from whatever cause they may originate, or however they may differ in minor respects, the states of the vital organs will be nearly similar in all." [23.] We may see how nearly the state of the circulation in the second stage resembles inflammation, and how easily, by obstruction taking place in the capillary arteries, the simple typhus may lapse into the inflammatory variety of the disease.

"The practitioner, (says our author) should be constantly on his guard from the commencement, and day by day should make the most scrupulous inquiries, that he may be enabled to arrest the very first appearances of inflammation in a vital quarter." 23.

We shall not extract our author's description of the inflammatory typhus, that we may allow ourselves more space to insert his new, and most masterly delineation of the congestive typhus. We consider that the state of inflammation is, in all cases, the effect, and not a cause of fever: The inflammation may indeed begin even at the first period of increased excitement; but then this can only happen from the capillaries of the inflamed parts being predisposed to obstruction from the smallest degree of increased action.

"In typhus, the brain or its membranes, the spinal chord or its coverings, the lungs, the pleura, the mucous membrane of the trachea, the stomach, the liver, the peritonæum, and the small and large intestines are the parts most liable to be attacked by an acute or sub-acute form of inflammation." 26. "The brain and its investing membranes being more subject to inflammation in typhus, than any other parts of the system." 28.

"Whenever, after an attack of typhus, there is a distinctly felt and fixed pain in the head, chest or abdomen, with great quickness of the pulse, dryness of the tongue, anxious breathing, much general oppression, the presence of the acute form of inflammation may be inferred. If there be little or no pain, and the pulse should become very frequent, the respiration more hurried, the tongue more parched and foul, and the general oppression greater, the approach of the sub-acute form may be apprehended." P.27.

We must, however, refer to the work itself for the description of typhus when complicated with acute or subacute inflammation of the various vital organs, with the morbid appearances formed after death; only remarking generally, that the reading of such descriptions should excite in us all, an ardent desire to emulate the unwearied diligence displayed by our author in detecting the latent varieties of disease in cases of fever. General ideas will avail little at the bed-side of a patient, whose life is on the balance, in an attack of acute fever. The season of the year, the constitution, the ten thousand other causes which modify the peculiar nature of individual cases, give birth to a variety in the actual state of the patient, the extent of which can only be expressed by stating the exact number of those individuals who have, at any time, been afflicted with fever; for as no two cases were ever exactly alike, each case, practically considered, becomes a separate disease. It is impossible, therefore, to describe all the states of disease which occur in nature; and thus the lives of our patients hang upon the acuteness, or rather the diligence with which we have made ourselves, practically acquainted, with the precise state of their organic complaints. Well might Celsus remark, that no physician could take care of many patients; at least of those whose lives are in danger! And it strikes us, in consequence, that the written descriptions of these varieties are likely to be of much more use to the describer, than to the reader; for it is only by describing the essential peculiarity of each case for himself at the bed-side, that the practitioner can become able to seize upon it on the instant, and snatch his patient from impending destruction. What multitudes have perished from this, too often, reprehensible want of discrimination in medical men! How often, for instance, have we reprobated physicians for saying, " she is in a consumption, and therefore must die." There are many curable complaints which destroy life under the sweeping influence of the word consumption; and thus also, on the contrary, in fever, he who can pen such decriptions as are found in Dr. A's volume, will assuredly be likely to save from the grave many of those who would have perished miserably, if under less favourable auspices.

### *The Congestive Typhus.*

" The open forms of fever, in which the heat and arterial action are equally developed, will be found the least dangerous; whilst those of an obscure congestive character, in which neither heat nor arterial action is equally developed, are the most perilous and unmanageable. In congestive cases, the local accumulations of blood in the veins obstruct from the beginning the common series of febrile phenomena, and there is, in consequence, either a total want of morbid heat, or a concentration of it in some particular parts of the body; whilst others are considerably beneath the natural temperature. It is the entire absence, or the partial presence of excitement, which constitutes the chief external distinction between the severest forms of the congestive typhus, as they all coincide in oppressing the functions or in deranging the structure of some important organ, by an almost stagnant accumulation of blood in some parts of the venous system'" 75.

"The attacks of the most dangerous forms of the congestive typhus are generally sudden, and marked by many remarkable symptoms:—An over-powering lassitude; feebleness of the lower limbs; deep pain, giddiness, or sense of weight in the encephalon; a dingy pallidness of the face; anxious breathing; damp relaxed, or dry withered skin; and those peculiar conditions of the temperature which have been mentioned above. The pulse is low, struggling and variable; the stomach irritable; frequently there is an inability to hold up the head; and the mind is more often affected with dulness, apprehension, or confusion, than with delirium. The whole appearance of the sick impresses the attentive practitioner with the idea, that the system in general, and the brain in particular, are oppressed by some extraordinary load. Both the manner and look of the patients undergo early and great alterations; sometimes they slowly drawl out their words, or utter them in a hasty, and yet imperfect mode, like people who slightly stammer when embarrassed. They not unfrequently seem as if stunned by a blow, half drunk, or lost in a reverie; and, at times, have the bewildered aspect of persons suffering under the first shock of an overwhelming misfortune. The eye is occasionally glary and vacant, without redness; but, at other times, it is heavy, watery, and streaked with blood, as if from intoxication or want of sleep. At the commencement, the pulse is often less altered, as to frequency, than might reasonably be expected, yet, in general, it becomes very rapid towards the close; the tongue is usually little altered in the first stage, but in the last it is frequently rough, foul, and brown; the bowels are mostly very torpid in the beginning, and the stools procured dark and scanty; whereas, in the advanced stage, the bowels are generally loose, and the stools copious and involuntary. Eructations are not uncommon at all times, and the epigastric region is often much inflated. On account of the general torpor, the secretions are diminished or suppressed; and, as justly remarked by Dr. Robert Jackson, the skin is often in that peculiar state, that if blisters be applied, they either do not act at all, or so defectively, as to leave an appearance as if the part had been slightly seared by a heated iron. Petechiæ in general appear earlier in these, than in any othet varieties of typhus; and in the last stage, there are sometimes gangrenous spots on the extremities, oozings of blood from the mouth and nostrils, and hæmorrhage from the bowels.

"There are conditions of the sensorium, voluntary powers, and præcordia, no less than of the respiration, pulse, and skin, which mark the progress or decline of such affections with the greatest certainty. If the stupor or delirium continue to increase with an augmentation of the oppression, if the respiration become more anxious, the pulse weaker and quicker, the skin colder, as well as more flaccid, and especially if the stools or urine be passed insensibly, the case will almost invariably terminate mortally. But, on the other hand, if the stupor or delirium should disappear, while the oppression obviously abates, and the respiration becomes easy, the pulse full and regular, with an universally warm skin, a favourable prognosis may generally be given. The abatement however, of the delirium or stupor, unaccompanied with the other favourable signs enumerated, is not at all to be depended on; for patients sometimes become rational and collected a few hours before death, and that even when the brain is in a state of irretrievable disease, as the two cases and dissections before given may serve to illustrate. It must always be recollected, that in examples of congestive fever, there is a singular disposition to relapse; so that a patient may grow very suddenly and seriously worse, when all the previous symptoms might have led us to form a sanguine opinion. The consideration of this truth, should make us pause before we give our prognosis, or at least teach us that, in the severer modifications of congestive fever, the patieut is not always perfectly safe, until he is perfectly recovered.

"There are, comparatively, milder forms of the congestive typhus, in some of which the patient walks about for a few days after the infection has begun to operate, and complains little, except of uneasiness of the head, loss of appetite, and languor, appearing rather paler than when in health. If strictly attended to, however, by a medical observer, a change may usually be remarked in his whole demeanour; he cannot so steadily command his attention as before, is not only restless during the day, but watchful at night, and soon betrays an absence of mind or loss of memory. At length, he becomes garrulous like a half drunken person, or talks inconsistently with his former views and character; after the lapse of another day or two, the mental confusion is most obvious to every one; he begins to be unsteady in his gait, and has a heavy intoxicated cast of the countenance. If carefully examined at this period, his tongue will be found white, his pulse small, quick, and perhaps irregular; his breathing hurried; his bowels slow; his skin rather hot about the trunk, but coolish and damp on the extremities. If the disease be allowed to proceed, without decided interruption, the hands shortly become very tremulous, and the confusion of mind passes into delirium; yet, there is still a want of regular excitement, demonstrated by the alternate flushings and paleness of the face, the feebleness of the pulse, the unequal state of the whole circulation, the coolness of the extremities, the partially concentrated heat of the trunk, and the laxity of the skin. Aural and visual deceptions succeed and force the patient into violent exertions, and every attempt to over-power him by coercion, tends to aggravate the delirium, and sink the strength. His tongue grows daily fouler, and his debility greater; he begins to pick the bed clothes, and at last petechiæ and subsultus tendinum appear. About this period, the general turbulence sometimes unexpectedly abates, and he may become so serene and rational, as to give some hopes that a favourable crisis has really taken place; but the calm is most frequently deceitful, being soon followed by an universal collapse, in which death occurs, mostly without much struggling. Several cases, nearly answering to the above description, have fallen under my notice, and I have found, that if opportunely and properly encountered, they may generally be subdued; but that if over looked, or improperly treated in the commencement, they will commonly baffle the best directed measures.

"There are yet other forms of congestive typhus, which, after a day or two of lassitude, are usually denoted by chilliness, nausea, short quick breathing, with frequent sighing, unpleasant sensations at the stomach—and also by a white tongue; deprivation of taste, irregularity of bowels, dark bilious excrements, pain and giddiness of the head, an alarmed or confused state of the mind, paleness of the face, dejection and langour of the countenance, inflation of the epigastric region, and great prostration of strength. An imperfect excitement is gradually developed, which rises and falls three or four times in the course of twenty-four hours. During the slight exacerbations of the fever, the skin is hot and dry in some places, especially about the præcordia; the face flushed; the pulse rapid; the breathing quickened almost to panting; the eye glossy; the countenance agitated; and the mind solicitous. These short paroxysms of fever passing away, the skin grows damp and relaxed, the face pale, the pulse less frequent and more undulating, the breathing slower, the eye duller, and the countenance and mind more serene. After some partial efforts of this nature, the excitement is sometimes fully emerged, the fever may put on a simple or an inflammatory character; but it more often advances, with frequent heats and chills, as an irregular one of congestion, and, if left to itself, most frequently destroys the patient, within the first two weeks of the attack, by cerebral or hepatic derangement, or suddenly suppresses life, by an unexpected engorgement of the brain, or some other vital organ. In such affections, there are occasionally distinct remissions, and likewise apparent translations of local oppression from one part to another. The remissions are commonly fallacious, and the translations are always to be dreaded, for, independently of the mischief which they may produce in the viscera affected, they denote a loss of the equilibrium and a general disorder in the circulating system, which are not easily corrected. The remarks which have been made, as to the prognosis, in the severer sorts of the congestive fever, are applicable to the forms now described; except that, in the latter, delirium is sometimes a favourable symptom when it is of the light imaginative kind, and when it occurs with evidences of returning regularity in the circulation and excitement." 81.

These engorgements of the venous system are more frequently found to affect the large vessels about the right side of the heart, the veins of the brain and liver, and afterwards those of the spleen and lungs. Congestive fevers are seldom ushered in by rigors. Is there a deficiency of electric matter in congestive fever? Dr. A. says,

"In some diseases of general torpor, attended with venous congestion and a deficiency of animal heat, I have known patients bear an accumulated force of the galvanic fluid with pleasure and advantage; whereas, in diseases of excitement, attended with an elevation of temperature, the slightest change was painful and prejudical, so that the galvanic fluid became a test, whether the system was in a preternaturally torpid, or excited state."

Dr. A. disavows the doctrine of critical days. What may be called critical discharges occur, but not on any particular days. The simple fever, however, has a tendency to decline after a number of days, but this is not the case with the inflammatory or congestive fever.

We now come to the discussion of Dr. Armstrong's treatment of fever; and on this subject we claim our reader's serious attention, for we have repeatedly seen, with the deepest regret, that Dr. A's treatment of fever has been so totally misrepresented, that he has been branded with the name of rash, whilst he is in reality cautious to the greatest degree; and has been almost accused of destroying the patients, whilst, as we have the best means of knowing, he practises, at the Fever Institution, as elsewhere, with a success, which has appeared to us, most worthy of being generally known. Prompt and decisive in the early stages of fever, we know that he seldom loses a patient when placed under his care at the commencement of the disease. In the visceral inflammations too, which mark the middle stages of fever, so accurately does he make the necessary diagnosis, that it is only when his patients are brought into the hospital, in an irremediable state of collapse, that, we had almost said, any deaths occur; but even in cases like these, by changing the decisive treatment of earlier stages, for a mode of practice more cautious and delicate than can be conceived by those who have not witnessed it, he guides his patients towards health with a skill, which is truly extraordinary. In making this statement, we do not wish to reflect upon the practice of other physicians. We speak but what we have seen, and we speak it, because, by statements made without due consideration, much discredit has been thrown upon an excellent mode of practice, to the great detriment of those afflicted.

In continuation of our design of giving a full description of simple fever, we shall dwell upon Dr. A's treatment of simple typhus. In all cases Dr. A. enjoins absolute rest from the onset. In the stage of oppression, he recommends emetics, followed by a large cathartic injection, and purgatives by the mouth in full doses, to overcome the prevailing torpor of the system. As the surface is cold he enjoins, with these remedies, the warm bath and plentiful internal dilution by means of tepid barley water, or thin gruel with the addition, in very old or debilitated habits, of small portions of weak wines. The apartment not to be below 56 or 60° of Fahrenheit's scale, as inflammations often follow exposure to cold air in the stage of oppression. By such means as these, the circulation is equalized, and the fever is either cut short, or it merges into he stage of excitement.

In this stage the treatment will of course be very different. Dr. Currie's plan by affusion of cold water, will frequently extinguish the fever during the first, second, or third day of the stage of excitement; but after the fourth day of this stage, it is seldom useful; the tepid affusions or bath (94 or 96°) being, thence forward, better adapted to the state of the constitution. The evening is the most proper time for using the cold affusions; indeed Dr. A. adopts, with some exceptions, the excellent directions given on this subject by Dr. Currie; the water never being used below 60°. With such precautions cold, applied in these ways, has never produced ill effects; in the stage of oppression, however, or of collapse its use may be, and has been fatal. Let it be remembered too, that the cold affusions are so generally efficacious only in the simplest forms of fever.

"Seldom less than four or five alvine evacuations should be daily produced during the stage of excitement in tolerably robust subjects" [109]; and to procure this effect, tolerably full doses of medicine must be prescribed. Thus, the intestines will be unloaded, healthy secretions be restored, irregular visceral distributions of blood removed, and the skin be relaxed. Care should be taken to prevent retention of urine. Wine is prohibited in all cases of the stage of excitement. The diet to consist solely of milk largely diluted with water, a little thin arrow root, milk-whey, barley water, or thin gruel. [113] These, with "the admission of fresh cool air, frequent changes of linen, thin bed-coverings, cold sub-acid drinks, quietness, and the abstraction of every extraordinary stimulus," [114]; form the sum of our author's treatment in the stage of excitement.

The stage of collapse is generally, in the milder cases of simple fever, the first approach towards health, and therefore little treatment is required; light nutriment will generally be all that is necessary. In more severe instances, laxatives are often alone required, unless in neglected cases attended with "great prostration of the natural powers, flushed face, suffused eye, delirium, or some degree of stupor, high breathing, foul tongue, and quick uneven pulse," [115]; "when full doses of brisk purgatives will bring away a load of fæces with evident relief. When, however, copious, black, bloody stools are passed without any offensive odour," [117], and the skin shews petechiæ like drops of very black ink, aperients will increase the consequent effusions of blood from the nostrils, mouth, bladder, or bowels, and cause a sudden depression of the vital powers. Fresh air, lemon juice mixed with a little Madeira and water, with very small doses of opium and aromatics, will in such cases form the best mode of treatment. The blood is evidently altered, and rendered unfit for the purposes of vitality. Would the exhilarating gases be useful?

We have but one observation to make on this treatment. Dr. A. it is true, recommends wine and other stimulants, as fresh malt liquor in the stage of collapse; but he by no means uses them liberally. Bark and other tonics, he apparently never recommends. Is this quite right? We feel that he acts upon the fact, that, in cases of death, in the stage of collapse, signs of previous or existing inflammations are found, at least, in the more inflammatory cases; but this can hardly be said to apply to the simple fevers, in which very slight or no marks of inflammation are found, and, as it is almost an universal mode of practice to precribe such remedies largely in the stage of collapse, and, therefore, we presume not a very fatal one; may we not ask, whether he has not somewhat overlooked this class of remedies in his practice? In the stage of collapse, cold air is studiously to be excluded from the patient's apartment. In the immense variety of states which occur in inflammatory fevers, Dr. A's chief reliance is placed upon the lancet. "In the beginning, or acmè, of the acute inflammation," let it be used vigorously: "but, if the topical affection has continued for some days, and there are symptoms of a present or an approaching collapse, let not the evidences of any local derangement induce the practitioner to hazard general venesection, as he values the life of the patient or his own reputation." [135] This is not the language of rashness, nor is it indiscriminate abuse of the lancet. Local blood-letting, however, is frequently of great use. But the greatest accuracy is required, to note the exact situation of the patient, and stage of his complaint; and it must not be forgotten, that fevers from the contagion of typhus "will not bear so large and repeated losses of blood, as simple acute inflammatory, such as gastritis, unconnected with contagion." [137]—Faintness, however, should be produced, and this by as small a loss of blood as possible, and therefore the patient should be bled standing, or with the trunk at least erect; "one or two moderate bleedings, followed by purgatives, blisters, leechings, or alteratives," [146] will be generally sufficient in the inflammatory typhus. Fifty-four ounces seem to have formed the largest quantity ever drawn by Dr. A. in inflammatory typhus, until he bled himself to fifty-five ounces, and Mr. Cavel, his pupil, to sixty ounces. Dr. A. speaks very highly of bleeding by leeches; he says that it produces a greater effect upon the heart than the same quantity of blood drawn in any other way would do. Blisters he never uses in the inflammatory fevers until evacuations have been premised; but they are then very serviceable. He does not use them also, "at a very late period of the disease, when the exhaustion is excessive, except where there is a tendency to coma." The cold affusions are not so efficacious as in simple typhus; but under proper circumstances, they may be applied after evacuations.

We must content ourselves with these short notices of the general treatment in the inflammatory fever; and must refer to the volume itself, and to the attentive consideration of our readers, for the proper modification of it, so as to meet the peculiarities of the inflammation, as it occurs in various organs.

### *Treatment of Congestive Typhus.*

In the congestive variety of typhus, there is much "apparent debility," but still the most efficacious remedies are bleeding, purging, and the warm bath. "It is in the first stage only of the highly contagious typhus that genera blood-letting is admissible." 181. The blood will often merely trickle at the commencement, being much darker and thicker than usual; whereas, after some time, it will be of a brighter colour, and flow freely, so as not to be so easily restrained as in ordinary cases. Where the congestion affects the head, the jugular vein or temporal artery should be opened. The pulse generally rises; sometimes, however, the reverse takes place, when the bleeding should not be repeated; but warm wine and water be given and the warm bath, at 100 degrees, impregnated with salt, be used.

It is necessary to give purgatives in large doses. "In such rapid cases, I have generally given a scruple of calomel at first, repeating much smaller doses, three or four times, on the first day of the attack" [185], "with jalap and very large stimulating enemata; and when the bowels have resisted their united influence, saline purgatives have been added, that no time might be lost" [186].—Patients almost invariably recover with rapidity, when ptyalism is excited; to favour which, Dr. A. adds to the calomel, after its purgative effect has been established, small doses of opium, antimony, and camphor. In the very severe cases, a "very powerful impression must be made within the first twenty-four hours, or little good in general can afterwards be effected; so rapid does the stage of collapse supervene, when the visceral congestions are not diminished soon after the attack." 188.

"Venesection is certain destruction when the general relaxation has occurred in the congestive typhus." 194. Stimulants may be moderately exhibited, both during the depletion and afterwards; blisters also, and after depletion, an antimonial emetic. It is clear to us, that Dr. A. is not himself quite satisfied with this mode of treating the congestive typhus; indeed, he says so in the volume before us. For ourselves, we confess that we think it defective; if we mistake not, the essence of the congestive state is debility; and the practical desideratum is to restore the lost balance of the circulation. The blood is black and the danger great, because the circulation is nearly at a stand; under a sudden attack of debility of the heart, that quantity of the blood which was easily circulated during health becomes destructive by its preponderance. We therefore find, with Dr. A. that lessening this quantity is often an essential part of the treatment. The bleeding, however, only acts mechanically: we consider the good of purging to be in like manner mechanical. The warm bath, or rather the hot bath, acts as a direct stimulus; so do blisters; emetics indirectly. According to these principles, may not the conjunction of general stimulants with the above remedies be considered as especially indicated? The case is, however, a very difficult one. The disease we consider to be induced by a sudden loss of vital, or, as we speak, nervous energy, produced by the contagion or other cause of fever. Now, more than a certain shock to the nervous system must necessarily be fatal; but it appears to us, that in less severe cases, the practical indication is, to stimulate the nervous energy, and assist its efforts to accomplish a reaction, by clearing away, as far as possible, the impediments which obstruct it in its struggle to overcome the load which oppresses it. Amongst other stimuli, Dr. A. suggests the employment of galvanism or electricity, and we think with great propriety. We hope he will himself put his suggestions to the test of experience.

We have thus given a very slight outline of the principles upon which Dr. A. describes and treats fevers, and our illustrations have been taken from his luminous account of typhus fever in its simple inflammatory and congestive forms.

We have done this, because Dr. A's Essay upon the common continued fever being itself only intended as illustrative of the tract on typhus, it comprehends only the peculiarities of that class of fevers, and therefore is not put into the form of a regular disquisition. But this is really of very little consequence; for, as Dr. A. has very justly remarked, however varied fevers may be, in their external symptoms, their internal pathology is invariably the same. The greatest differences between them, consist chiefly in their degrees of violence; fevers from contagions, however, vary from common continued fevers in some essential particulars; and therefore it would be very advantageous to be able always to discriminate between them. The scarlet fever, the small-pox, and measles, indeed, can be easily distinguished; but the diagnosis between typhus and the common continued fever is often very difficult. Still, it would seem, that the operation of contagion upon the human body, is, in all cases, very peculiar; and, therefore, we may expect, that future observation will enable us to distinguish the two diseases in all cases. At present, we find that typhus differs from the continued fever, in being more violent in its forms and symptoms; in apparently running a certain course, at least when once fairly established; in the greater prostration of the bodily powers, and in the fluids of the body being altered in their constitution. By the fluids, we mean the blood, from which all the other fluids, except the contents of the absorbent system, are secreted. The blood, in measles and small-pox, is evidently altered; for we believe that it will communicate those diseases by inoculation; and that some change takes place in the blood in typhus fever also, is proved by that remarkable duskiness which pervades the skin so early; and which, towards the end of the disease, becomes apparent, even in the muscles and other solid parts. Would inoculation with this dark blood produce typhous fever? Perhaps it might; as, indeed, we can only explain the production of contagion, by supposing it to be secreted from the blood. With such ideas of contagion, it will be anticipated, that we do not consider it probable, that common continued fever can take on the properties of typhus, and become contagious. We have witnessed much inconclusive reasoning on this subject, and with regret, as the question is one of great importance. We hold, that a peculiar assemblage of physical phenomena forms the origin of every disease. Some of these assemblages occur very frequently; hence the corresponding disease is frequently generated; others are of more rare occurrence, and the consequent disease is more rarely produced. Some have the peculiar property of producing a disease which propagates itself by what we all call contagion; others do not give this power. The latter class of diseases can only be produced in one way; whilst, in the case of contagious diseases, we think, that there are two modes of production; the one by propagation from body to body, that is by contagion: the other, by being generated anew by the accidental meeting together of the physical phenomena, the assemblage of which first gave birth to them. Some of these assemblages so rarely happen, that the disease, the small-pox for instance, is absolutely unknown in certain climates; whilst other contagious diseases occasionally appear without our being able, in the slightest degree, to trace them to contagion. For these reasons, amongst others, we think it very unphilosophical to argue that continued fever is convertible into typhus, except, indeed, by supposing that the state of common continued fever forms one part of the peculiar assemblage of physical phenomena necessary for the production of typhus.

We see that Dr. A. agrees generally with the opinion here expressed; in page 299, he says,

"Now, in the course of my experience, I cannot recollect a single instance where a fever proceeding from such ordinary causes, (cold, &c.) was changed into one possessing the specific contagion of typhus; and therefore I am strongly disposed to imagine, that the thing never happens."

Again, in the same page,

"Have we any other example in medical literature, where an ordinary disease, not having the property of contagion at the beginning, is converted, during its progress, into a specific disease, having the property of contagion?"

We are aware that the present opinion of many practical men is, in a great degree, contrary to this; but we are somewhat unwilling to place implicit confidence in the suggestions of experience, as at present constituted. We know too little of the phenomena of febrile diseases, of the the difference produced in disease, by its various causes, or of the mode in which fever is produced; in short, we are too essentially ignorant of fever, in all its details, for us to be competent judges of almost any case which can be brought before us. For instance, we have heard of one most experienced practitioner, who went abroad a staunch believer in something like the opinions which we have here expressed. The yellow fever (we believe) broke out in his ship, under such peculiar circumstances as completely to satisfy his mind that it was contagious. We much doubt whether, after he recollects the various instances which have occurred, of yellow fever being confessedly produced by miasmata from a ship's hold, he will be able to satisfy his mind, that this disease might not have been propagated in the same way.* Practitioners are also inclined to argue, from the common opinion, that contagious fevers always run a certain course, that such course is essential to the fever; and, therefore, if cases of typhus are even cut short by treatment, which they often are, they infer, that such cases mark a difference between the contagion of typhus, and that of other ad-

---

* As the opinions here maintained, respecting contingent contagion, are at variance with those uniformly promulgated in this Journal, some *inconsistency* may be complained of. But it will readily be perceived that we put no restraint on the sentiments of our Reviewers, although we steadily maintain our own, which, indeed, are diametrically opposite to those both of Dr. Armstrong and his Reviewer, on the point under discussion. Ed.

mitted contagious fevers, as the small-pox, &c. This argument is a very dangerous one to the holder. In the first place, is the course of contagious fevers so determinate? Has hooping cough, for instance, a determinate course? Has not our settled opinion, on that head, prevented us from attempting to cut them short? Were we to witness a case of small-pox now, we should attack it much more vigorously, at the commencement, than we formerly did; and we cannot say what would be the effect upon the duration of the disease, after deducting the necessary time for the local disorder to run its course; so, therefore, have not we improperly attributed the time physically necessary for the resolution of eruptions of contagious fevers, to the effect of the contagion? and consequently, would not the small pox frequently be cut short, if unattended by an eruption which generally requires a certain time for its resolution?

In the next place, if a fever, confessedly from typhus contagion, have ever been cut short (and who will doubt that such has been the case) in the first stages, such an argument must prove that typhus is not contagious at all. If so, the plague is not contagious; for we believe that that disease has often been cut short; and we believe also, that its course is not a determinate one; at least, both diseases are similarly situated, and must stand or fall by the same argument.

We have entered thus largely on the subject of contagion, firstly, to show our general ignorance of the essential properties of contagion, and thus, secondly, to call the attention of practitioners to the study of the phenomena which attend contagion in its operation. Are the first stages of any contagious fevers contagious? or, when do they begin to be so? How long do persons continue to emit contagious matter after the febrile symptoms have ceased, in scarlet fever for instance? These are a few of the questions to which answers are required. Were we to speculate, we should ask, if there are not many contagious fevers which have not a determinate course? if, in those which have, it is not produced from an accidental and unessential circumstance? If contagions are not secretions from the diseased blood; if fevers ever become contagious until the blood has, in the progress of the disease, taken on the peculiar change required; if a common fever is convertible into a contagious fever, without the occurrence of further circumstances—if a contagious fever can be produced in other than two ways; viz. by contagion, or by the re-occurrence of that peculiar assemblage of natural phenomena from which it first arose.

The subject of fever has been much canvassed lately, from the existence of the present widely-spread epidemic, which has been so destructive in various parts of the united kingdom. From Dr. A's connexion with the fever Institution, he has, of course, had great opportunities of investigating its true nature; and it would appear from his observations, that much of the contrariety of character which has appeared in the description of different practitioners, has arisen from the epidemic having been formed of three distinct descriptions of fever, namely, "typhus, the common continued fever, and a low fever of irritation, arising from filth and defective nutriment." 316.

The following extract from Dr. Cleverly's Annual Report will show under what unfavourable circumstances Drs. Cleverly and Armstrong have had to combat an epidemic, the common character of which has been extremely severe, during the whole term in which they have practised in the Fever Institution.

"The present epidemic commenced in London, about the end of March, 1817, and during twelve or fourteen months the proportion of deaths, in comparison with former years, was certainly very small. In May last, however, the mortality began to be more considerable, and the physician, at that time officiating, reported, that the disease 'seemed to have assumed a character of greater severity, and that the mortality had, in consequence, been very considerable.' And in the report for June, the same physician declared, that the mortality had been unprecedented in any former reports of this Institution; and this he ascribed, in part, to the increased virulence of the malady, but principally 'to the very advanced state of the disease in which many of the patients were admitted, precluding the possibility of affording them effectual relief.' And, in fact, the mortality in June was 1 in $4\frac{1}{2}$, which is greater than at any period (with a single exception) since the Institution has existed. In July (about the middle of which the present physicians were appointed) the ratio was as 1 to about $5\frac{1}{10}$, and the average ratio for the whole period comprehended in the present report is nearly as 1 to 6. Epidemics have always been observed to differ much from each other in the severity of their characters, and even from themselves, in different periods of their course; so that, in all Institutions, the mortality, in different years, has been found to vary greatly; and, indeed, in the London House of Recovery, it has fluctuated between 1 in $12\frac{1}{2}$ and 1 in $3\frac{3}{4}$. But however various this result, we know that the disease was treated by the same physician, in the several instances alluded to, and with the great care and talent by which he is distinguished.

"Besides, the mortality in the wards of this Institution cannot be considered as representing, at any time, the general destructiveness of the disease, or the success with which it may be treated in its early stages; for the House has become the receptacle for a very considerable number of the worst cases of typhus in its most advanced stages; and to this cause, certainly, the great mortality is to be attributed. It will appear, on examination, that a very large proportion of the patients has been sent into the House, from under the care of general practitioners, and from the medical officers attached to parishes; and this often not till the disease had assumed its most alarming and desperate character. Patients themselves often evince considerable reluctance to leave their homes, however miserable, and seek relief in an hospital; and, when the first dread of the epidemic is over, they are frequently retained by their relations, till the aspect of the disease has alarmed these for their own safety.

"From these causes, it has arisen, that patients have often died before the porters could arrive at their dwellings; that others have expired in the house before the physicians could see them; and that others, again, with cold extremities, livid, and senseless, have survived their reception only a few hours; and, had it been possible, that many of the patients could have left their fever at the gates, they must still have died, from the great mischief which important or vital organs had sustained during the first period of its destructive and, perhaps, unrestrained violence."*

We hope no person will say that we have praised the labours of Dr. A. too highly, until they have first read this volume as well as his other works; we say so on their own accounts, for we venture to affirm, that no man of sound judgment and right feelings can fail to find in them such profound marks of industry, of caution, of acuteness, of philosophy, and of benevolence, as must win from him his most unqualified praise. We have proved that we do not think the work perfect, by the unreservedness of our animadversions; but we felt that we were discussing the points in question with a friend, who would more gladly be shown the defective parts of his arguments, than be praised for their general or particular excellence.

We shall conclude, by saying, it has repeatedly struck us, that the character of Dr. Armstrong, as a physician and as a man, bears considerable resemblance to that of his great countryman and favourite, SYDENHAM. We all feel a species of affection for Sydenham, when we consider his uprightness, his simplicity of heart, and his philanthropy; whilst our admiration is similarly excited, in

* The seventeenth Report of the Institution for the Cure and Prevention of Contagious Fever in the Metropolis; For 1819. London. Printed for the Institution.

contemplating the pure spirit of philosophy, the talent for observing natural phenomena, and the freedom from doctrinal bias, which so distinctly pervade the writings of both. In both of them we find a proneness to theory, but in both an undeviating adherence to facts in practice; in both an almost faulty mildness and forbearance of manner; but in both, an honest inflexibility in the expression of truth. We prophecy, too, that the fate of each will be the same; that increasing years will bring to Dr. Armstrong an increase of dignity and comfort, as we believe they did to his predecessor; and that, hereafter, the memory of both will be cherished by posterity, with similar feelings of reverence and respect.*

* The unexpected length of this article has induced us to print the remainder of this number on a small type, so that we may compress as much matter as possible within the usual limits. Ed.

